# Cross cultural adaptation and validation of the Malay Kidney Disease Quality of Life (KDQOL-36™)

**DOI:** 10.1186/s12882-019-1397-8

**Published:** 2019-06-20

**Authors:** Kent Ka Kian Goh, Pauline Siew Mei Lai, Soo Kun Lim

**Affiliations:** 1 Department of Primary Care Medicine, University Malaya Primary Care Research Group (UMPCRG), Faculty of Medicine, University of Malaya, 50603 Kuala Lumpur, Malaysia; 2 Department of Medicine (Nephrology) Faculty of Medicine, University of Malaya, 50603 Kuala Lumpur, Malaysia

**Keywords:** Malay, Hemodialysis, Kidney disease quality of life, Validity, Reliability; health related quality of life

## Abstract

**Background:**

In Malaysia, the prevalence of chronic kidney disease is high (9.1%). To date, no questionnaire that specifically assesses the health-related quality of life of patients with chronic kidney disease has been validated in Malaysia. Malay is the national language of Malaysia and spoken by the majority of its citizens. Therefore, the aim of our study was to cross-culturally adapt and validate the Malay Kidney Disease Quality of Life-36 (KDQOL-36) among patients with chronic kidney disease.

**Methods:**

The English version of the KDQOL-36 was translated according to international guidelines to Malay. Content validity was verified by an expert panel and piloted in five patients. Our instrument was then administered to patients with chronic kidney disease stage 1-3A and patients on hemodialysis at baseline and 4 weeks later.

**Results:**

A total of 181/232 patients agreed to participate (response rate = 78.0%). The majority were male (69.6%) with a median age of 51.0 years. Exploratory factor analysis found that the KDQOL-36 had three domains. All three domains showed low to moderate correlation (Spearman’s Rho = 0.297–0.610) with the Europe Quality of Life Five Dimension questionnaire. Patients on hemodialysis (physical component summary = 39.8; mental component summary = 53.1;burden of disease = 37.5; symptoms/burden list = 75.0; effects of kidney disease on daily life = 68.8) had significantly worse quality of life than patients with chronic kidney disease stage 1-3A (physical component summary = 49.9; mental component summary = 52.9; burden of disease = 75.0; symptoms/burden list = 85.4; effects of kidney disease on daily life = 93.8, *p* < 0.001) except for the mental component summary. This indicates that the Malay KDQOL-36 has achieved adequate known-groups validity. Cronbach alpha ranged from 0.872–0.901, indicating adequate internal consistency. At retest, intraclass correlation coefficient ranged from 0.584–0.902, indicating moderate to good correlation.

**Conclusion:**

The Malay Kidney Disease Quality of Life-36 was found to be a valid and reliable tool to assess the quality of life in patients with chronic kidney disease. This tool can now be used to assess the health-related quality of life (HRQOL) in patients with chronic kidney disease, as HRQOL is an important independent predictor of patient outcome.

**Electronic supplementary material:**

The online version of this article (10.1186/s12882-019-1397-8) contains supplementary material, which is available to authorized users.

## Background

Globally, chronic kidney disease (CKD) is one of the important causes of mortality and morbidity [1]. More than ten million people worldwide have CKD which may progress to end stage renal disease (ESRD) [2]. In 2014, the prevalence of CKD in the United States was 13.6%. A 2016 review showed that the prevalence of CKD was higher in developed countries (such as the United States, Europe and Canada) than in economically developing countries (such as sub Saharan Africa and India) [3]. This may be due to the prevalence of higher dietary risks, body mass index (BMI), systolic blood pressure and co-morbid conditions in developing countries [3]. In Malaysia, the prevalence of CKD was 9.1% in 2011 [4]. In 2015, there were 37,183 patients receiving dialysis in Malaysia with 7597 new patients for dialysis [5].

Health-related quality of life (HRQOL) is a marker for burden of disease which can be used to assess the effectiveness of a treatment and predict the risk of adverse outcomes [1]. HRQOL is the patient’s subjective perception of their illness and treatment with regards to their physical, psychological and social-well-being [6]. Patients on dialysis have significant symptom burden and impaired quality of life, as they have a high number of comorbidities [7, 8]. In a study conducted in Hong Kong, patients on dialysis had a symptom burden of at least 9 symptoms (mean = 9.3 + 4.7) with fatigue (75.4%), cold aversion (68.7%), pruritus (65.7%), lower torso weakness (59.7%) and difficulty in sleeping (61.9%) as the most prevalent symptoms [8]. Dialysis patients with sleep disturbance was associated with lower HRQOL [9]. Therefore, assessing HRQOL of patients with ESRD is important as it is an independent predictor for patient outcomes.

A generic quality of life (QOL) tool (e.g. SF-12) is designed to assess the function and well-being of individuals regardless of their specific condition [10]; whilst a “disease targeted” HRQOL instrument assesses QOL in specific disease conditions [10]. The most comprehensive method for assessing QOL would be to include both generic and disease targeted content in the instrument [10]. Several tools such as Kidney Disease Questionnaire (KDQ) [11], Kidney Transplant Questionnaire (KTQ) [12] and Netherlands Cooperative Study on Adequacy of Dialysis (NECOSAD) [13] have been developed for assessing HRQOL in patients with CKD [14]. Among these tools, we selected the Kidney Disease Quality of Life (KDQOL) as it was developed for individuals with kidney disease who may or may not be on dialysis [15]. The KDQOL also has adequate to excellent internal consistency [16]. The Kidney Disease Quality of Life-36 (KDQOL-36) is an instrument that consists of both a generic core (SF12) and disease specific components [10]. However, the KDQOL-36 has not been validated in Malaysia. When adapting questionnaire, the cultural, idiomatic, linguistic and contextual aspects concerning its translation should be considered [17]. Therefore, the cross-cultural adaptation of a health status self-administered questionnaire in a different language requires a unique method to reach equivalence between the source and target version of the questionnaire through the process of translation, adaptation and assessment of validity and reliability of the targeted questionnaire [17]. It was important for us to validate the KDQOL-36 in Malay as Malay is the national language of Malaysia and spoken by the majority of its citizens. In addition, the prevalence of CKD in Malaysia was 9.1% [4].

Therefore, the aim of this study was to cross-culturally adapt and validate the Malay Kidney Disease Quality of Life among patients with chronic kidney disease.

## Methods

### Translation of the English kidney disease quality of life (KDQOL-36™) to Malay

Permission to use the KDQOL-36 was obtained from the original developer (via email on 25 June 2016). Translation of the English KDQOL-36 to Malay was performed according to international guidelines (Additional file 1) [18].

### Face and content validity

Face validity is defined as “the degree to which (the items of) an health-related-patient-reported outcome (HR-PRO) instrument indeed looks as though they are an adequate reflection of the construct to be measured” [19]. Content validity is defined as “the degree to which content of an HR-PRO instrument is an adequate reflection of the construct to be measured” [19] Face and content validity of the Malay KDQOL-36 was assessed by an expert panel (consisting of a nephrologist, an academician experienced in the validation of instruments and a pharmacist). A pilot study was then conducted on five patients with CKD stage 1-3A. Two participants were confused with item no. 3 (“climbing several flights of stairs”) as they were unsure whether the item meant “climbing several flights of stairs” or just “several steps”. In Malay, “climbing several flights of stairs” or just “several steps” are expressed in the same way. Hence, for this item, the researcher had to explain to each participant that this item meant “climbing several flights of stairs”.

### Validation of the Malay kidney disease quality of Life-36

This validation study was conducted at the Nephrology clinic in a tertiary hospital and its affiliated dialysis centers located in Kuala Lumpur from July 2016 to July 2017.

### Participants

Known-groups validity is demonstrated when a test or questionnaire can discriminate between two groups known to differ on the variable of interest [20]. Previous studies showed that HRQOL progressively declined across the stages of CKD [21–23]. Hence, two groups of participants were recruited so that known-groups validity could be assessed. We hypothesized that the HRQOL of patients on hemodialysis would be worse than patients with CKD stage 1-3A.

### Patients on hemodialysis (patient group)

Patients > 21 years of age, who could understand Malay and were on hemodialysis for at least 3 months were recruited. Patients with mental disabilities were excluded.

### Patients with chronic kidney disease stage 1-3A (control group)

Patients > 21 years of age, who could understand Malay and with CKD stage 1-3A (defined as glomerular filtration rate (eGFR) value of 45 to > 90 mL/min/1.73 m^2^ with evidence of kidney damage) were recruited. Patients who were diagnosed with rheumatoid arthritis, active cancer and mental disabilities were excluded.

### Sample size

Sample size was calculated based on the number of items to participant ratio of 1:5 to perform factor analysis [24]. There are 36 items in the KDQOL-36. Therefore, the minimum number of participants required was 36*5 = 180.

### Baseline demographic form

A baseline demographic from was used to collect participants’ baseline demographic data and other relevant information.

### The Malay kidney disease quality of life 36

The KDQOL-36 is a self-administered tool which measures kidney disease-related HRQOL. The original tool consists of 134 items, and was too lengthy to administer in clinical practice [25]. Thus, a shorter version – the KDQOL-SF™ version 1.3 was developed. It consists of 36 items, and two cores: the SF-12 (i.e. generic QOL) and the disease-specific core. The generic core consists of two domains: the physical component summary (PCS) [6 items] and the Mental Component Summary (MCS) [6 items]. The disease-specific core consists of 24 items with 3 domains: symptoms and problems (12 items), burden of kidney disease (4 items) and effects of kidney disease (8 items). The raw scores were transformed according to the scoring manual [26] ranging from 0 to 100, where a higher score indicates better QOL. Patients that were not on hemodialysis were not required to answer item 28a “problems with your access point”.

### The Malay EuroQol 5 dimensions questionnaire (EQ-5D-5L)

Convergent validity is the extent of different instruments to measure the same construct and that correlates with each other [27]. The Malay EQ-5D-5 L was used to assess the convergent validity of the KDQOL-36. The EQ-5D-5L consists of 5 items (which assesses five dimensions: mobility, self-care, usual activities, pain/discomfort and anxiety/depression) and a visual analog scale (EQ-VAS) [28]. The response for the five items was a 5-point Likert scale where 1 indicated better QOL, whilst 5 indicated poorer QOL. Scores were converted to 0 to 100%. The EQ-VAS requires patients to rate their own health using a scale which ranged from 0 (worst imaginable health) to 100 (best imaginable health).

### Data collection

Convenience sampling was used to recruit participants. Potential participants were approached, and the purpose of the study was explained to them. For those who agreed to participate, written informed consent was obtained. At baseline, participants were asked to fill the baseline demographic form, the Malay KDQOL-36 and the EQ-5D-5L. One month later, participants were asked to answer the Malay KDQOL-36 again.

### Data analysis

Data was analyzed using the Statistical Package for Social Science (SPSS) version 20.0 software (Chicago, Illinois, USA). Normality was assessed using the Kolmogorov Smirnov test. Non-parametric tests were used as data was not normally distributed. Descriptive statistics were used to describe the demographic data of participants. Categorical variables were presented using percentages and frequencies, whilst continuous variables were presented using median and interquartile ranges.

### Validity

Validity is defined as “the degree to which HR-PRO instrument measures the construct(s) it purports to measure” [19].

### Factor analysis

The dimensionality of the Malay KDQOL-36 was analyzed using exploratory factor analysis (EFA). Principal factor analysis and promax oblique rotation was used as the domains were correlated [29]. The cut-off point for the factor loadings was 0.4. [30].

### Convergent validity

Convergent validity is defined as “the degree to which scores of a measure associate with scores on other measures that are intended to assess similar construct” [17]. The score for the three domains of the KDQOL-36 were compared with the scores of the EQ 5D5L and VAS. Correlations were calculated using Spearman’s rho coefficient: < 0.20 shows a very weak correlation, 0.20–0.40 shows weak correlation, 0.40–0.70 shows moderate correlation, 0.70–0.90 shows strong correlation and > 0.90 shows very strong correlation [31].

### Known-groups validity

The Mann-Whitney-*U*-test was used to determine whether the Malay KDQOL-36 was able to discriminate between patients with stage 1-3A CKD (eGFR > 90–45 ml/min/1.73m^2^) and patients undergoing dialysis.

### Reliability

Reliability is defined as “the degree to which the measurement is free from measurement error” [19] In the reliability section, we analyzed the data of both patients with stage 1-3A and patients on hemodialysis as a whole.

### Internal consistency

Internal consistency is defined as “the degree of interrelatedness among the items” [19]. Internal consistency was assessed using Cronbach’s alpha coefficient to determine the extent that all items in a test measures the same concept [32]. This was done for the entire instrument, and for the different domains. Cronbach’s alpha < 0.70 have inadequate consistency; 0.70–0.90 suggests adequate internal consistency [32]. Corrected item-total correlation was also performed. Corrected item-total correlation > 0.4 is considered acceptable [33]. The effect of removing an item on Cronbach’s alpha was also determined.

### Test-retest

The intra-class correlation coefficient (ICC) was used to analyze responses obtained at test and retest. Values > 0.9 indicate excellent reliability, 0.75–0.90 indicate good reliability; 0.5–0.75 indicate moderate reliability and < 0.5 indicate poor reliability [34].

## Results

**Fig. 1:**
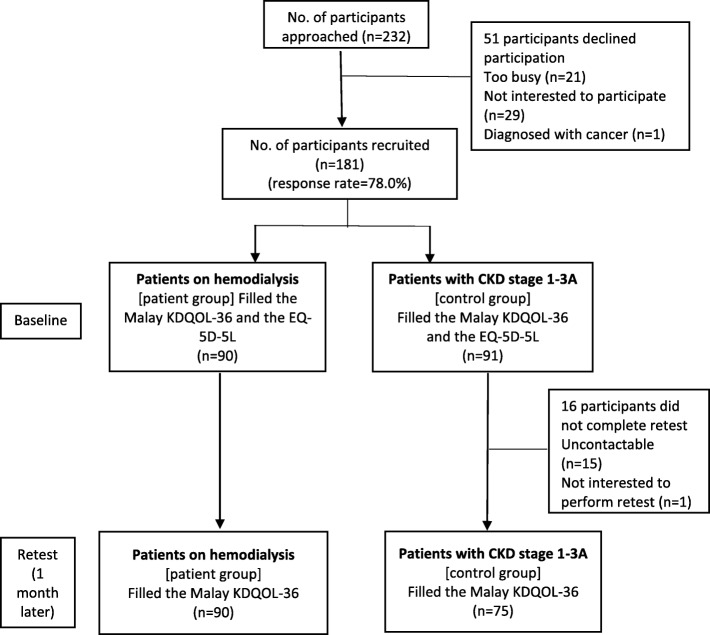
Algorithm of how participants were recruited

A total number of 181/232 agreed to participate (response rate = 78.0%) [Fig. 1]. The demographic characteristics of participants are shown in Table 1. The majority were male (69.6%) with median age of 51 years (Table 1).

**Table 1:** Demographic characteristics of participants

	Total (*n* = 181) N (%)	Patients with CKD (Control group (*n* = 91) N (%)	Patients on hemodialysis [Patient group] (*n* = 90) N (%)	Chi square / z-value	*p*-value
Gender					
Male	126 (69.6)	70 (76.9)	56 (62.2)	4.623	0.032*
female	55 (30.4)	21 (23.1)	34 (37.8)		
Median age in years (IQR) [range]	51 (41–61.5)	54 (39–66)	50.5 (41.75–58.0)	−1.345	0.178
< 30 years	7 (3.9)	6 (6.6)	1 (1.1)	19.374	0.002*
31–40 years	36 (19.9)	19 (20.9)	17 (18.9)		
41–50 years	44 (24.3)	17 (18.7)	27 (30.0)		
51–60 years	39 (21.5)	12 (13.2)	27 (30.0)		
61–70 years	48 (26.5)	31 (34.1)	17 (18.9)		
> 70 years	7 (3.9)	6 (6.6)	1 (1.1)		
Median BMI in kg/m^2^ (IQR) [range]*	25.5 (22.4–28.7)	26.6 (24.1–31.3)	23.9 (20.6–27.9)	−3.205	0.010*
Ethnicity					
Malay	82 (45.3)	52 (57.1)	33.3 (30)	14.897	0.02*
Chinese	69 (38.1)	23 (25.3)	51.1 (46)		
Indian	24 (13.3)	14 (15.4)	11.1 (10)		
Others	6 (3.3)	2 (2.2)	4.4 (4)		
Level of education					
Primary school (6 years of education)	7 (3.9)	3 (3.3)	4 (4.4)	19.227	> 0.001*
Secondary school (11 years of education)	71 (39.2)	26 (28.6)	45 (50)		
Diploma/ (12 years of education)	48 (26.5)	21 (23.1)	27 (30)		
Tertiary (> 12 years of education)	55 (30.4)	41 (45.1)	14 (15.6)		
Presently Working					
Working	83 (45.9)	51 (56)	32 (35.6)	7.650	0.006*
Income (calculate USD equivalent as your manuscript will be read internationally)					
< 238.41 USD$	61 (33.7)	21 (23.1)	40 (44.4)	29.865	> 0.001*
238.41 USD$-476.82 USD$	42 (23.2)	14 (15.4)	28 (31.1)		
476.82 USD$-715.23 USD$	24 (13.3)	17 (18.7)	7 (7.8)		
715.23 USD$-953.64 USD$	14 (7.7)	8 (8.8)	6 (6.7)		
953.64 USD$-1192.05 USD$	13 (7.2)	12 (13.2)	1.1 (1)		
> 1192.05 USD$	26 (14.4)	19 (20.9)	7 (7.8)		
Duration patient has been diagnosed with CKD in years; median [IQR]	5.0 (2.0–10.3)	4.0 (2.0–10.0)	7.0 (3.0–11.3)	−2.129	0.033*
= < 10 years	134 (74.0)	69 (75.8)	65 (72.2)		
11–20 years	34 (18.8)	13 (14.3)	21 (23.3)		
21–30 years	7 (3.9)	3 (3.3)	4 (4.4)		
31–40 years	2 (1.1)	2 (2.2)			
> 40 years	1 (0.6)	1 (1.1)			
Duration on dialysis in months; median [IQR]	–	–	36 (24–84)		

*BMI* Body Mass Index, *CKD* Chronic Kidney Disease, *ESRD* End Stage Renal Disease, *IQR* Interquartile range, *USD* United States Dollars

### Construct validity

#### Factor analysis

EFA found that the Malay KDQOL-36 was a 3-factor model (Table 2). The scree plot for each domain is provided in Additional file 2.

**Table 2:** Exploratory factor analysis of the Malay kidney disease Quality of Life-36

Item no.	Description of items	Subscale	Factor loadings			KMO	AVE	CR
			1	2	3			
How true or false is each of the following statements for you?								
13	Kidney disease interferes too much with my life	Burden of kidney disease	0.892			0.785	0.698	0.902
14	Too much time is spent dealing with my kidney disease		0.888					
15	I feel frustrated dealing with my kidney disease		0.765					
16	I feel like a burden on my family		0.791					
	During the past 4 weeks, to what extent were you bothered by each of the following?	Symptom/burden list						
22	Shortness of breath			0.691		0.875	0.427	0.890
25	Washed out or drained			0.744				
27	Nausea or upset stomach			0.633				
23	Faintness or dizziness			0.700				
26	Numbness in hands or feet			0.683				
17	Soreness of muscle			0.690				
18	Chest pain			0.635				
20	Itchy skin			0.673				
21	Dry skin			0.659				
19	Cramps			0.587				
24	Lack of appetite			0.448				
	Some people are bothered by the effects of kidney disease on their daily life, while others are not. How much does kidney disease bother you in each of the following areas?	Effects of kidney disease on daily life						
32	Your ability to travel				0.766	0.892	0.502	0.889
34	Stress or worries caused by kidney disease				0.746			
30	Dietary restriction				0.727			
36	Your personal appearance				0.719			
29	Fluid restriction				0.717			
31	Your ability to do work around the house				0.717			
33	Being dependent on doctors and other medical staff				0.670			
35	Your sex life				0.593			

*KMO* Kaiser-Meyer-Olkin test, *AVE* average variance extracted, *CR* composite reliability

### Convergent validity

The scores for the three domains in the KDQOL-36 were found to be significantly correlated to the EQ-5D-5L. However, the association between the KDQOL-36 and EQ-5D-5L was weak to moderate. The association between the domains “burden of disease”, “signs/symptoms list”, “effects of kidney disease on daily life” with the EQ-5D-5L was − 0.456, − 0.610 and − 0.588 while the association these domains with the EQ VAS was 0.297, 0.434 and 0.361, respectively.

### Known-groups validity

Patients on hemodialysis (physical component summary = 39.8; mental component summary = 53.1; burden of disease = 37.5; symptoms/burden list = 75.0; effects of kidney disease on daily life = 68.8) had significantly worse quality of life than patients with chronic kidney disease stage 1-3A (physical component summary = 49.9; mental component summary = 52.9; burden of disease = 75.0; symptoms/burden list = 85.4; effects of kidney disease on daily life = 93.8, *p* < 0.001) except for the mental component summary, indicating that the Malay KDQOL-36 has achieved adequate known-groups validity (Table 3).

**Table 3:** Known-groups validity of the Kidney Disease Quality of Life −36

			Mann Whitney-U test	
Domain	Patients on hemodialysis (patient group) median (IQR)	Patients with CKD stage 1-3A (control group) median (IQR)	z-score	*p*-value
Physical component summary (PCS)	39.8 (33.4–46.4)	49.9 (37.0–54.0)	−4.456	< 0.001*
Mental component summary (MCS)	53.1 (43.5–57.9)	52.9 (47.0–58.2)	−0.437	0.662
Burden of disease	37.5 (25.0–6.3)	75.0 (62.5–87.5)	−8.015	< 0.001*
Symptoms/burden list	75.0 (66.7–83.3)	85.4 (75.0–89.6)	−4.719	< 0.001*
Effects of kidney disease on daily life	68.8 (53.1–81.3)	93.8 (84.4–100.0)	−8.542	< 0.001*

*IQR* Interquartile range

### Reliability

The overall Cronbach alpha of the Malay KDQOL-36 was 0.715. Cronbach’s alpha values for the domain ranged from 0.872–0.901. At test-retest, the ICC of the KDQOL-36 showed moderate to good correlation (ICC = 0.584–0.902) (Table 4).

**Table 4:** Psychometrics of the Malay Kidney Disease Quality of Life-36

Domain	No.	Item	Cronbach alpha	Corrected item-total correlation	Intra-class correlation
Burden of kidney disease	13	Kidney disease interferes too much with my life	0.901	0.826	0.808
	14	Too much time is spent dealing with my kidney disease		0.821	0.841
	15	I feel frustrated with my kidney disease		0.725	0.824
	16	I feel like a burden on my family		0.746	0.885
Symptoms/ burden list	17	Soreness of muscle	0.872	0.607	0.753
	18	Chest pain		0.553	0.584
	19	Cramps		0.506	0.790
	20	Itchy skin		0.533	0.732
	21	Dry skin		0.534	0.802
	22	Shortness of breath		0.680	0.819
	23	Faintness or dizziness		0.657	0.806
	24	Lack of appetite		0.428	0.635
	25	Washed out or drained		0.678	0.674
	26	Numbness in hands or feet		0.608	0.777
	27	Nausea or upset stomach		0.660	0.779
	28a	Problems with your access site (HD patients only)		0.347	0.825
Effects of kidney disease on daily life	29	Fluid restriction	0.884	0.666	0.864
	30	Dietary restriction		0.677	0.761
	31	Your ability to do work around the house		0.674	0.786
	32	Your ability to travel		0.716	0.883
	33	Being dependent on doctors and other medical staff		0.624	0.707
	34	Stress or worries caused by kidney disease		0.693	0.861
	35	Your sex life		0.553	0.902
	36	Your personal appearance		0.683	0.782

## Discussion

The Malay KDQOL-36 was found to be a valid and reliable tool to assess the HRQOL of patients with CKD in Malaysia.

EFA found that the Malay KDQOL-36 was a 3-factor model: “burden of kidney disease”, “symptoms/burden list” and “effects of kidney disease on daily life”. Our findings were similar to a Singaporean study which reported that the KDQOL-36 was a three-factor model [35]. However, the authors of this study used confirmatory factor analysis to confirm the number of factors, whereas we used EFA. We were not able to analyze our data using CFA, as the minimum sample size required to conduct CFA was 315 [36].

The Malay KDQOL-36 was able to discriminate between patients who were on hemodialysis and early stage (CKD stage 1-3A) in all domains except for the mental component summary. In a previous study, the HRQOL of patients were discriminated based on subgroups of demographic data of patients of the study [37]. The study showed that being female, unemployed, having history of hospitalization during the past 6 months, and being on a longer duration of hemodialysis had worse HRQOL [37]. At present, no other study has assessed the discriminative validity of the KDQOL-36 using patients at different stages of CKD. [23].

The scores from the three domains of the Malay KDQOL-36 were significantly correlated to the EQ-5D-5L and EQ VAS score, which was similar to a previous study [38]. The correlation in our study was negative because for the KDQOL-36, as a higher KDQOL-36 score indicates a better QOL, whilst a higher score in EQ5D5L indicates a worse QOL.

The overall Cronbach alpha of the Malay KDQOL-36 was 0.715, whilst the Cronbach alpha of the individual domains ranged from 0.872–0.901, which was similar to a previous study [38]. At test-retest, ICC values ranged from 0.584–0.902, which was lower compared to previous studies [37, 38]. This was due to item no. 18 (“in the past 4 weeks, to what extend were you bothered by chest pain?”) where two participants selected the answer “not at all bothered” at test (Likert scale = 1), whilst at retest they answered, “extremely bothered” (Likert scale = 5). These patients may have experienced chest pain during the period between test and retest.

One of the limitations of our study was that we were unable to perform CFA as the minimum sample size required to perform this analysis was 315. Another limitation was that our patients were recruited using convenience sampling, and may not be representative of the general population [39].

## Conclusion

The Malay KDQOL-36 was found to be a valid and reliable tool to assess the HRQOL in patients with CKD. This tool can now be used to assess the HRQOL in patients with chronic kidney disease, as HRQOL is an important independent predictor of patient outcome.

## Additional files

Additional file 1: Translation of the English Kidney Disease Quality of life (KDQOL-36) to Malay. (DOCX 39 kb)

Additional file 2: Scree plot for exploratory factor analysis of burden of disease, symptoms/burden list and effects of kidney disease on daily life for the Malay KDQOL-36. (DOCX 42 kb)

## Data Availability

The datasets used and/or analyzed during the current study are available from the corresponding author on reasonable request.

## Abbreviations

CFA: Confirmatory factor analysis
CKD: Chronic kidney disease
EFA: Exploratory factor analysis
eGFR: Glomerular filtration rate
EQ-5D-5L: Europe quality of life-5-dimension-5-level
EQ-VAS: Europe quality of life-visual analog scale
ESRD: End stage renal disease
HR-PRO: Health-related-patient-reported outcome
HRQOL: Health-related quality of life
MCS: Mental component summary
PCS: Physical component summary
QOL: Quality of life
