# Supplementary material for: Cross cultural adaptation and validation of the Malay Kidney Disease Quality of Life (KDQOL-36™)
Source: BMC Nephrol. 2019 Jun 20;20:226. doi: 10.1186/s12882-019-1397-8 (PMC6585031; doi:10.1186/s12882-019-1397-8)
Supplement: Supplementary file 1 — Translation of the English Kidney Disease Quality of life (KDQOL-36) to Malay. (DOCX 39 kb) [file 12882_2019_1397_MOESM1_ESM.docx]

Appendix 1: Translation of the English Kidney Disease Quality of Life-36 (KDQOL-36) to Malay

KDQOL-36 English

Forward translation 2

(version 1b)

Forward translation 1

(version 1a)

Expert panel met to produce the final version of the forward translation

(version 2)

Backward translation 2

(version 3b)

Backward translation 1

(version 3a)

Expert panel met to produce the final draft of the Malay KDQOL-36

(version 4)

Pilot tested (n=5)
