# Supplementary material for: Cross cultural adaptation and validation of the Malay Kidney Disease Quality of Life (KDQOL-36™)
Source: BMC Nephrol. 2019 Jun 20;20:226. doi: 10.1186/s12882-019-1397-8 (PMC6585031; doi:10.1186/s12882-019-1397-8)

Appendix 2: Scree plot for exploratory factor analysis Malay KDQOL-36

Figure 1: Scree plot for exploratory factor analysis of burden of disease of the Malay KDQOL-36


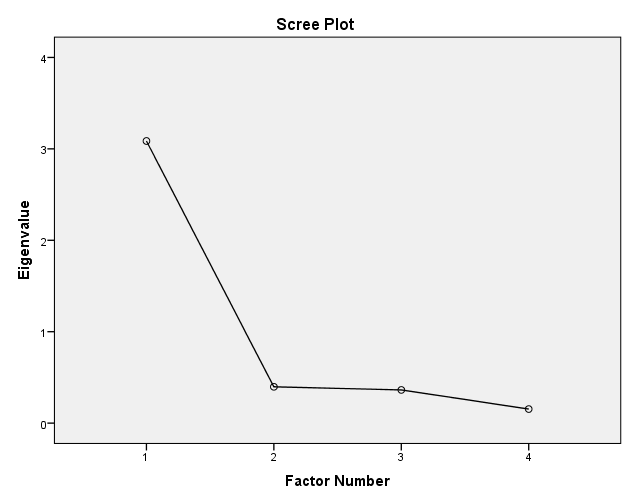


Figure 2: Scree plot for exploratory factor analysis of symptoms/burden list for the Malay KDQOL-36


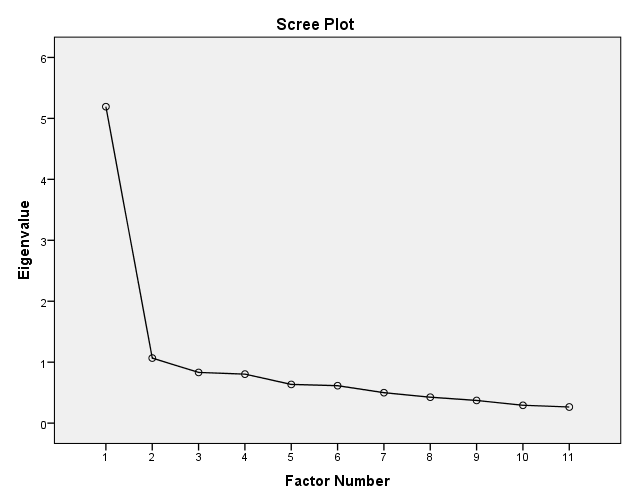


Figure 3: Scree plot for exploratory factor analysis of effects of kidney disease on daily life for the Malay KDQOL-36


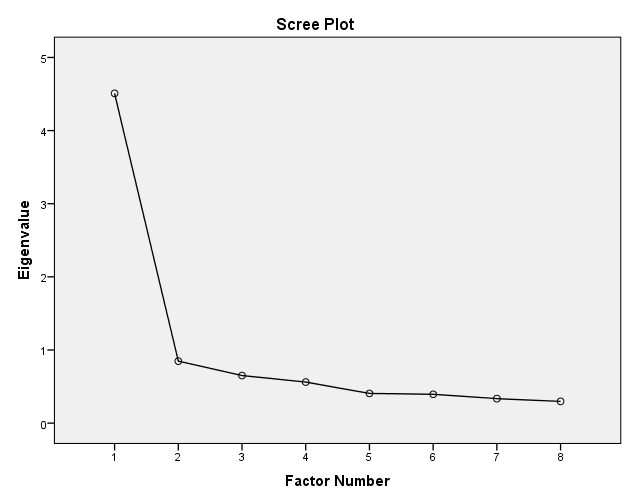

Supplement: Supplementary file 2 — Scree plot for exploratory factor analysis of burden of disease, symptoms/burden list and effects of kidney disease on daily life for the Malay KDQOL-36. (DOCX 42 kb) [file 12882_2019_1397_MOESM2_ESM.docx]
